# 

**Published:** 1891-12

**Authors:** 


THE JOURNAL learns and is pleased to announce that Dr. B. E. Hadra has returned to Galveston and permanently located.

---

WE CLUB WITH

The Cosmopolitan ($3.50) including Memoirs of Lee or Grant (70c) for .... $5.70.
The Texas Sanitarian ($2) at .... 3.00
The Mississippi Med. Journal ($2) at .... 3.00
Bacteriological World ($2) at .... 3.25
Kansas City Med. Index ($2) at .... 2.50
N. O. Med. and Surg. Journal .... 3.50
Dr. King's Stories of a Country Doctor at .... 2.75

---

SUBSCRIBE FOR

DANIEL'S TEXAS MEDICAL JOURNAL FOR 1892.

Contains only original matter, and is the favorite and most popular journal in the South. As an advertising medium, it is unsurpassed. Send for rates. Agents wanted to canvass for subscriptions in Texas. Liberal commissions.

